# The antiplatelet agent revacept prevents the increase of systemic thromboxane A_2_ biosynthesis and neointima hyperplasia

**DOI:** 10.1038/s41598-020-77934-x

**Published:** 2020-12-08

**Authors:** Sara Alberti, Qianqian Zhang, Ilaria D’Agostino, Annalisa Bruno, Stefania Tacconelli, Annalisa Contursi, Simone Guarnieri, Melania Dovizio, Lorenza Falcone, Patrizia Ballerini, Götz Münch, Ying Yu, Paola Patrignani

**Affiliations:** 1 Department of Neuroscience, Imaging and Clinical Science, “G. D’Annunzio” University, Chieti, Italy; 2 CAST (Center for Advanced Studies and Technology) (Ex CeSI-MeT), “G. D’Annunzio” University, Via dei Vestini 31, 66100 Chieti, Italy; 3 International Peace Maternity and Child Health Hospital of China Welfare Institution, Shanghai, China; 4 Department of Innovative Technologies in Medicine and Dentistry, “G. D’Annunzio” University, Chieti, Italy; 5 AdvanceCOR GmbH, Martinsried, Germany; 6 Shanghai Institute for Biological Sciences, Chinese Academy of Science, Shanghai, China; 7 Department of Pharmacology, School of Basic Medical Sciences, Tianjin Medical University, Tianjin, China

**Keywords:** Pharmacology, Cardiovascular biology

## Abstract

Neointima hyperplasia is a crucial component of restenosis after coronary angioplasty. We have hypothesized that enhanced generation of platelet-derived thromboxane (TX)A_2_ in response to vascular damage plays a critical role in neointimal hyperplasia and that antiplatelet agents may mitigate it. In cocultures of human platelets and coronary artery smooth muscle cells (CASMC), we found that platelets induced morphologic changes and enhanced the migration of CASMC. The exposure of platelets to Aspirin [an inhibitor of cyclooxygenase (COX)-1] reduced the generation of TXA_2_ and prevented the morphological and functional changes induced by platelets in CASMC. Platelet-derived TXA_2_ induced COX-2 and enhanced prostaglandin (PG)E_2_ biosynthesis in CASMC, a known mechanism promoting neointimal hyperplasia. COX-2 induction was prevented by different antiplatelet agents, i.e., Aspirin, the TP antagonist SQ29,548, or Revacept (a dimeric soluble GPVI-Fc fusion protein). The administration of the novel antiplatelet agent Revacept to C57BL/6 mice, beginning three days before femoral artery denudation, and continuing up to seven days after injury, prevented the increase of the systemic biosynthesis di TXA_2_ and reduced femoral artery intima-to-media area and the levels of markers of cell proliferation and macrophage infiltration. Revacept might serve as a therapeutic agent for percutaneous coronary angioplasty and stent implantation.

## Introduction

Percutaneous transluminal coronary angioplasty (PTCA) with or without vascular stenting is commonly used for the treatment of coronary heart disease^1^.

However, restenosis occurs in 30–50% of patients undergoing simple balloon angioplasty, and in 10–30% of patients who receive an intravascular stent^1^.

Collagen exposure at the vascular damage site leads to platelet adhesion and aggregation mainly via the Glycoprotein (GP) VI signaling^2^. Thromboxane (TX)A_2_, a primary product of arachidonic acid metabolism in activated platelets via the cyclooxygenase(COX)-1 pathway, promotes migration and proliferation of vascular smooth muscle cells(VSMC)^3–5^. TXA_2_ operates through the binding to TXA_2_ receptors (TP)^6^, which by coupling to Gα 12/13 lead to YAP/TAZ activation; they are the major downstream effectors of the Hippo signaling pathway^7^. Injury-induced vascular proliferation is reduced in mice lacking the TP receptor or treated with a TP antagonist^8^. In patients undergoing PTCA, enhanced systemic biosynthesis of TXA_2_ (as assessed by measuring the urinary levels of the major enzymatic metabolites, TXM) was largely suppressed by low-dose Aspirin^9,10^, thus suggesting that this biochemical change reflects platelet activation at the site of vascular damage^10^.

Restenosis is an excessive response of the coronary artery to damage during angioplasty. It consists of platelet activation, inflammatory cell recruitment, VSMC proliferation, and migration to the intima. VSMC change their phenotype from contractile to synthetic, which promotes the extracellular matrix synthesis^1^. These events constitute the neointimal hyperplasia, which contributes to postprocedural lumen loss. In animal models, peak neointimal growth is observed 28 days following bare-metal stents placement, whereas in humans, it is identified at 6–12 months^11^.

COX-2, an inducible enzyme that mediates the generation of prostanoids in inflammation^3^, plays a role in restenosis progression^12^. COX-2 is markedly upregulated in vascular inflammation, such as atherosclerosis and balloon-injured arteries^13,14^. Pharmacological inhibition or specific deletion of COX-2 in VSMC reduces vascular neointima hyperplasia in response to mechanical injury^12,14^. COX-2 mediates these effects by enhancing the generation of prostaglandin (PG)E_2_ that contributes to vascular restenosis pathogenesis through the activation of the PGE_2_ receptor subtype EP3α/β and its signaling pathways cAMP/protein kinase A and phosphatidylinositol 3-kinase^12^. Moreover, genetic disruption of microsomal PGE_2_ synthase-1 attenuates neointima formation after vascular injury^15^. However, the pharmacological inhibition of vascular COX-2 by the use of selective COX-2 inhibitors (named coxibs) is not recommended in this setting for their cardiovascular hazard^16^.

In the early phase of restenosis, platelets are the chief cellular players^17^ through their adhesion to VSMC, at the site of the vascular damage, facilitated by subendothelial collagen exposure, and to the release of soluble factors, such as lipid mediators derived from arachidonic acid (especially TXA_2_)^18^.

Revacept, a novel antiplatelet agent in clinical development, is a soluble form of the platelet GPVI receptor that binds specifically to collagen at vascular damage sites, thus inhibiting platelet adhesion and aggregation without affecting general hemostasis in humans^19^. Using fluorescence-labeled glycoprotein VI (GPVI)-Fc in ApoE-deficient [ApoE^(−/−)^-mice, fed with a 1.25% cholesterol diet over 16 weeks], the mechanical-induced carotid injury was associated with increased GPVI-Fc-binding to injured carotids compared to intact carotids^20^.

In cocultures of human platelets and coronary artery smooth muscle cells (CASMC), we addressed the hypothesis that platelet-derived TXA_2_ is the trigger of the induction of COX-2-derived-PGE_2_, a pathway involved in the development of neointimal hyperplasia^12^. Then, we tested whether Revacept attenuates neointimal formation and prevents enhanced systemic TXA_2_ biosynthesis in mice after arterial injury. Our findings suggest that the inflammatory response to vascular damage after coronary angioplasty might be mitigated by avoiding platelet-vessel wall interaction and preventing enhanced TXA_2_ biosynthesis using Revacept.

## Methods

### Coculture experiments with human coronary smooth muscle cells (CASMC) and washed human platelets isolated from venous blood

CASMC (at passage 5) (Lonza Walkersville Inc, MD, USA) were cultured in flasks coated with collagen type I from rat tail (BD Bioscience Discovery Labware, Bedford MA, USA), at 37 °C in a humidified atmosphere of 5% CO_2_ in air, in the culture system BulletKit (Lonza) [containing SmBM (Basal Medium) and SmGM-2 (Smooth Muscle Cell Growth Medium-2) SingleQuots supplements] and 5% fetal bovine serum (FBS). Then, CASMC (0.8 × 10^5^ cells) were seeded in a six-multiwell plate coated with collagen type I from rat tail in 2 ml of culture system containing 5% FBS. After 48 h (h), the medium was changed with Dulbecco’s modified Eagle’s medium (DMEM) containing 0.75% bovine serum albumin (BSA) (Sigma-Aldrich, Milan, Italy) and 1% polymyxin-B sulphate (Sigma-Aldrich) and human washed platelets (0.5 × 10^8^ cells in 100 μl), freshly isolated from concentrated buffy coats (obtained from the blood bank of SS Annunziata Hospital, Chieti, Italy), as previously described^21^, were added. This study was carried out following the recommendations of the Declaration of Helsinki. Healthy volunteers (23–45 years) who had not taken any nonsteroidal antiinflammatory drug (NSAID) in the 2 weeks before blood donation were enrolled. Informed consent was obtained from each subject. All experimental protocols were approved by the Blood Center (Asl2 Lanciano-Vasto-Chieti, Italy), local Ethics Committee of "G. d’Annunzio" University of Chieti-Pescara. Separate experiments (n) were performed using different buffy coats.

### Immunofluorescence

CASMC (0.8 × 10^5^ cells) were cultured alone or cocultured with platelets (0.5 × 10^8^) for 8 h; then, cells were washed twice with phosphate buffer solution (PBS) pH 7.4 and fixed in acetone/methanol (40:60) for 20 min at room temperature. Cells were blocked with a filtrated solution of BSA (3%) in PBS for 30 min at room temperature. Cells were incubated overnight at 4 °C with polyclonal antibodies anti-COX-1 or anti-COX-2 (1:200, Cayman Chemical, Ann Arbor, MI, USA) and anti-α-SMA (1:200, Santa Cruz Biotechnology, Dallas, Texas, USA). Cells were then washed three times with PBS and incubated with the secondary antibodies, donkey anti-rabbit Alexa Fluor 488 and goat anti-mouse Alexa Fluor 546 (1:1000, Life Technologies, Waltham, MA, USA) for 1 h at room temperature. Cells were washed three times with PBS and incubated for 5 min with 4′,6-diamidino-2-phenylindole (DAPI; 300 nM, Sigma Aldrich) to label nuclear DNA. Finally, cells were washed and mounted in slides with Diamond antifade mounting media (Life Technologies). Slides were observed with a Zeiss LSM 510 meta microscope (Carl Zeiss, Jena, Germany), and confocal images were analyzed using Zeiss LSM 5 series software (Carl Zeiss).

### Pharmacological treatments in vitro

The selective COX-1 inhibitor Aspirin (acetylsalicylic acid, ASA, Sigma-Aldrich), the selective COX-2 inhibitor Rofecoxib (Witega Laboratorien, Berlin, Germany), and the TP antagonist SQ 29,548 (Sigma-Aldrich) were dissolved in dimethylsulfoxide (DMSO, Sigma-Aldrich). Rofecoxib (0.3 μM) or SQ 29,548 (10 μM), or DMSO were added to CASMC for 30 min before the addition of platelets. In other experiments, platelets were pretreated with Aspirin (100 μM) or DMSO before the incubation with CASMC; briefly, platelet-rich plasma was incubated with Aspirin (100 µM) or DMSO for 30 min at room temperature; then platelets were isolated^21^, washed twice, and either cultured alone or cocultured with CASMC for 20 h. Finally, Revacept^19^ or vehicle [PBS/4% mannitol/1% sucrose (Sigma Aldrich)] were added to CASMC for 1 h, and then, the medium was changed, replaced with the platelet cell suspension, and incubated for 20 h.

CASMC or platelets cultured alone or cocultured were lysed in 1%Triton and 1 mM phenylmethylsulphonyl fluoride (Sigma-Aldrich) and stored at − 80 °C until assayed by Western blot. In some experiments, the supernatants were collected, centrifuged at 700×*g* for 5 min, and stored at − 80 °C until assayed for prostanoid levels.

### Migration assay

Cell migration of confluent CASMC cultured alone or with platelets, pretreated, or not with Aspirin, was evaluated by scrape wounding assay, as previously described^22,23^. Briefly, after 20 h of platelet-CASMC cocultures, platelets were washed away. Cells, seeded on a 6-multiwell dish, were scraped using a 200 μl pipette tip, simulating a wound, incubated in DMEM 0.75% BSA, and monitored periodically by light microscope up to 24 h. Image processing was performed using Image J 1.44 software (NIH, USA), and the percentage of cell-free area and cell covered area were calculated using the analysis particle tool.

### Biochemical analyses

TXB_2_ and 6-keto-PGF_1α_ [the nonenzymatic hydrolysis product of TXA_2_ and prostacyclin (PGI_2_), respectively], PGD_2_, PGE_2_, and PGF_2α_, were measured in cell culture media by previously described and validated immunoassay techniques^23,24^.

### Western blot analyses

Cell lysates were mixed with sodium dodecyl sulfate (SDS)(Sigma-Aldrich) sample buffer and heated to 95 °C for 5 min. Proteins (15 μg) were analyzed by SDS polyacrylamide gel electrophoresis (SDS-PAGE)^21,23^. Anti-COX-2, anti-COX-1 polyclonal antibodies (1:1000, Cayman Chemical), GAPDH polyclonal antibody (1:1000, Santa Cruz Biotechnology), or anti-β-actin polyclonal antibody (1:1000, Santa Cruz Biotechnology) were used^21,23^. Quantification of optical density (OD) of different specific bands was carried out using Alliance 4.7 and Alliance 1 D software (UVITEC, Cambridge, UK) and normalized to the OD of β-actin or GAPDH.

### Animals

Male C57BL/6 mice (8–10 weeks old, n = 20) were purchased from Shanghai SLAC Laboratory Animal Co., Ltd. (Shanghai, China). All animals were maintained and used according to the veterinary guidelines of the Institutional Animal Care and Use Committee of the Institute for Nutritional Sciences, Chinese Academy of Sciences, Shanghai (China). Experimental protocols were approved by the Institutional Animal Care and Use Committee of the Institute for Nutritional Sciences, Chinese Academy of Sciences, Shanghai (China). Every effort was made to minimize the number of animals used and any pain or discomfort experienced by the animals.

### In vivo study design and surgical procedures

The femoral artery wire injury model was described previously elsewhere^25,26^. A contralateral (left femoral arteries) sham surgery was used as a control by performing the protocol without the wire injury. Right femoral arteries were exposed by blunt dissection and subjected to denudation. Briefly, arteries were exposed by blunted dissection, dilated by topical application of one drop of 1% lidocaine hydrochloride (Sigma-Aldrich) and transverse arteriotomy was performed in the muscular branch; a straight spring wire (0.38 mm in diameter, COOK, Bloomington, IN, USA) was carefully inserted into the femoral artery for more than 5 mm toward the iliac artery and left in place for 1 min to denude and dilate the artery. Then, the wire was removed, and blood flow in the femoral artery was restored. Animals were randomly assigned to two different treatment groups. One group (n = 10) received Revacept, the protein obtained by fusing the extracellular domain of GPVI with the human immunoglobulin (IgG) Fc domain^19^ (at the dose of 2 mg/kg/day), via tail-vein injection, for three days before bilateral femoral artery denudation was performed. The treatment was continued up to day 10. Control group mice (n = 10) were treated with recombinant human IgG1 Fc (at the dose of 2 mg/kg/day) (Sino Biological Inc., China) via tail-vein injection. The compounds were dissolved in a solution of PBS/mannitol(4%)/sucrose(1%). The concentration of Revacept and Fc were selected based on previously published studies^27^. For all surgical procedures, the animals were ventilated with isoflurane and oxygen to maintain anesthesia throughout the experiment, and anesthetic depth was assessed by observing the reflex responses to paw pinches. Histology and immunohistochemistry were performed on specimens collected at day 32 (i.e., 28 days after surgery). Moreover, 24 h-urine collections were performed on day 0 (baseline), day 3, day 7, and 32 (i.e., before injury and 3 and 28 days after injury, respectively) for the assessment of 11-dehydro-TXB_2_ (TXM) levels, as a marker of the systemic TXA_2_ biosynthesis, mainly derived from the activity of platelet COX-1^28,29^.

### Histology and immunohistochemistry

At the end of the in vivo experiments (i.e., 28 days after surgery), all animals were euthanized and intracardially perfused with 10 ml of 4% paraformaldehyde (PFA, Sigma Aldrich). Femoral arteries were fixed with 4% PFA for 10–15 min and then embedded in paraffin. Paraffin-embedded Sections (7 μm) were stained with hematoxylin and eosin (HE, Sigma-Aldrich), and the intima-to-media area (I/M) ratio was assessed as previously described^12^. Briefly, the intimal area is the area encircled by the internal elastic lamina minus lumen area. The medial area is calculated as the area encircled by the external elastic lamina minus intima area. The intima-to-media ratio was calculated as the intimal area divided by the medial area. For immunohistochemistry, tissue sections were incubated with primary antibodies against Ki67 (1:500, Abcam, Cambridge, UK), CD68 (1:200, AbD Serotec, Kidlington, UK), followed by incubation with horseradish peroxidase(HRP)–conjugated secondary antibody [anti-rabbit IgG (Cell Signaling Technology, Leiden, The Netherlands)1:2000; anti-rat IgG (ProteinTech Group, Rosemont, IL, USA) 1:2000]. After three washings with PBS, samples underwent 3′-Diaminobenzidine (DAB, Sigma-Aldrich) staining, hematoxylin restaining, dehydration, and cover glass mounting. Slides were observed with a Zeiss Axiovision microscope (Carl Zeiss) and Image J 1.44 software was used for the analysis.

### Assessment of urinary levels of TXM

Two-hundred μl of 24 h-urine collection samples were extracted, and the levels of the TXM, i.e., an enzymatic urinary metabolite of TXB_2_, were measured by previously validated immunoassay techniques^28,29^. The urinary TXM levels were corrected for creatinine excretion and reported as ng/mg of creatinine.

### Statistical analysis

All values were reported as mean ± SD (standard deviation) in the text, while shown as mean ± SEM (standard error of the mean) in the Figures; the n values (indicating the number of separate experiments) were reported in Figure legends. Statistical analysis among two groups was performed by Student's t-test; for 3 and more groups, ANOVA (one-way or two-way) or Mixed-effects analysis for repeated measures (when some values were missed) were carried out using GraphPad PRISM software (version 8.00 for Windows; GraphPad, San Diego, CA, USA). *P* values < 0.05 were considered statistically significant.

## Results

### Effects of platelets on CASMC phenotype and morphology in vitro

**Figure 1:**
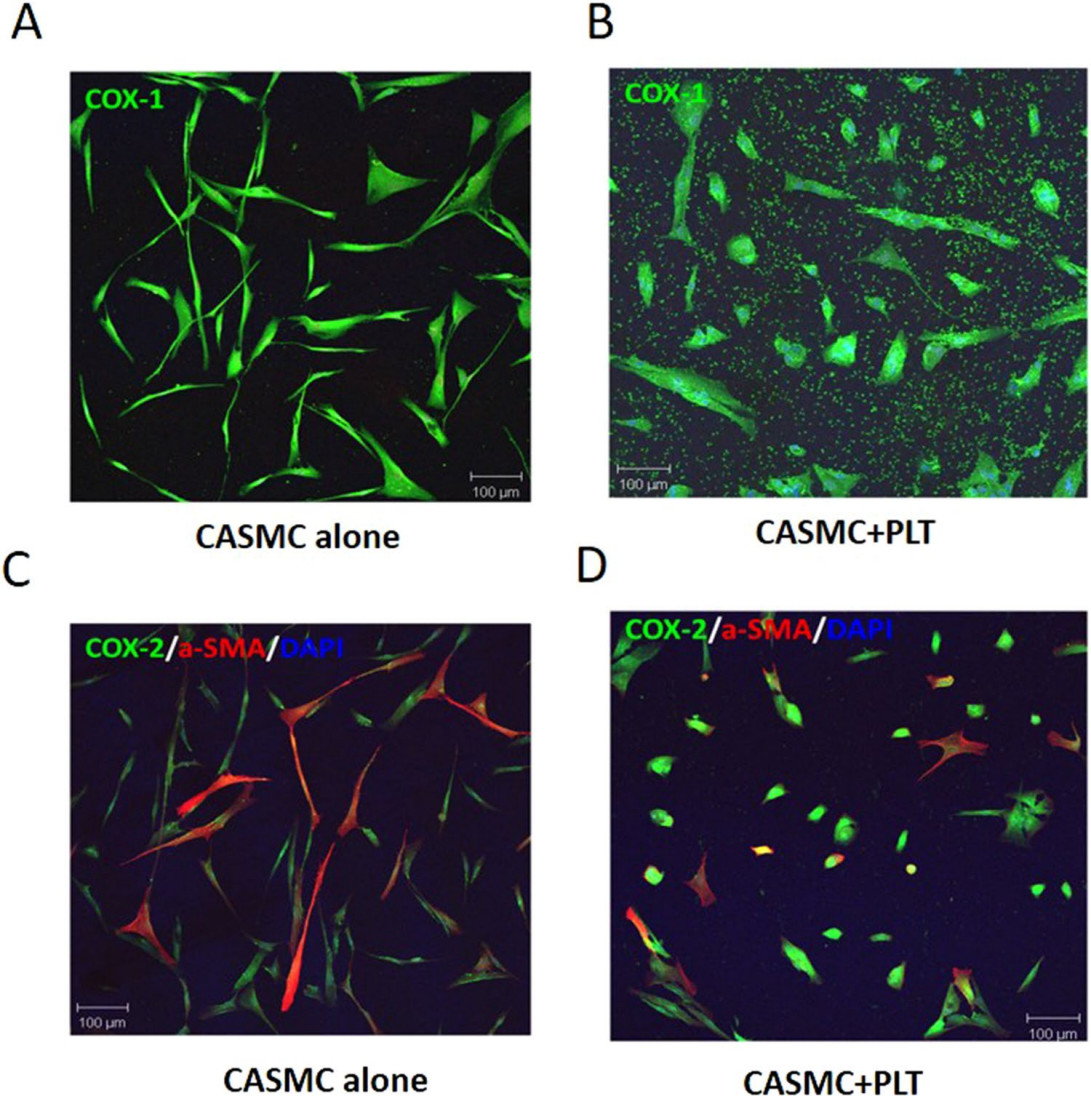
Effects of platelets on phenotypic modulation of CASMC. Human CASMC (0.8 × 10^5^ cells) were cultured alone or cocultured with human platelets (0.5 × 10^8^). COX-1 (green) (**A**,**B**) or COX-2 (green) and α-SMA (red) (**C**,**D**) expression was assessed by confocal microscopy. Image magnification was 10X.

**Figure 2:**
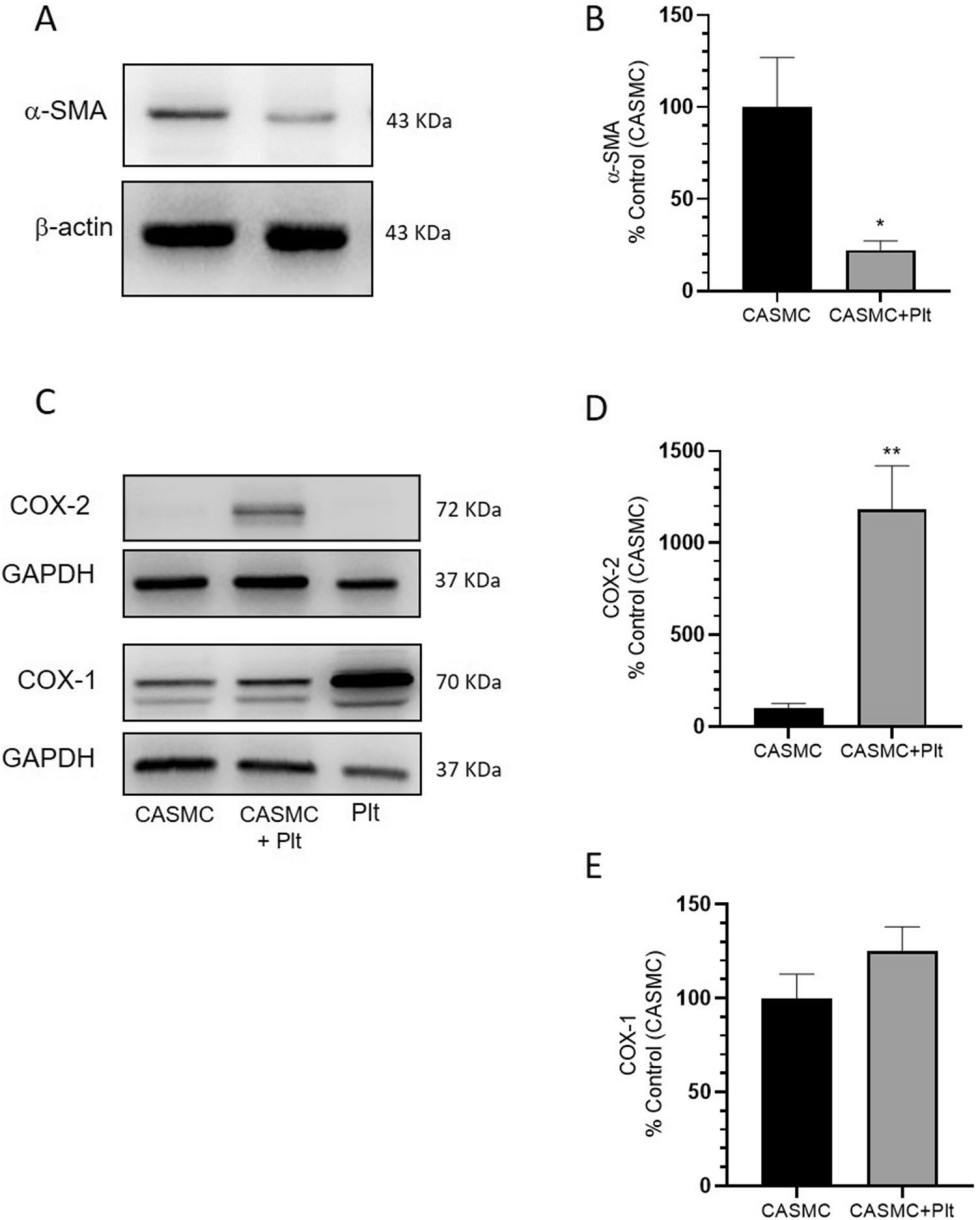
Effects of platelets on α-SMA, COX-2, and COX-1 protein levels in CASMC cultured alone or cocultured with platelets. (**A**) α-SMA levels were evaluated by Western blot in CASMC cultured alone or cocultured with platelets for 24 h; (**B**) the protein bands were quantified, and the optical density (OD) was calculated using laser densitometry and normalized to the OD of β-actin; data are reported as % of control (CASMC cultured alone), mean ± SEM, n = 5 (separate experiments); *P < 0.05 versus CASMC cultured alone (using Student’s t-test**)**. (**C**–**E**) COX-2 and COX-1 levels were evaluated by Western blot in platelets (Plt) or CASMC cultured alone or in CASMC cocultured with platelets (CASMC + Plt) for 20 h; (**D**,**E**) the protein bands were quantified and OD was calculated and normalized to the OD of GAPDH, data are reported as % of control (CASMC cultured alone), mean ± SEM, n = 5 and n = 3 (separate experiments), respectively; (**D**) **P < 0.01 versus CASMC cultured alone (using Student’s t-test**)**.

The changes induced by platelets on the morphology and the phenotype of the CASMC were assessed using confocal microscopy by the labeling of COX-1(a constitutive protein) and α-SMA [typical of a contractile phenotype]^30^. CASMC cultured alone appeared as spindle-shaped cells (Fig. 1A). The co-incubation with platelets led to epithelioid cell morphology, yielding a cobblestone pattern^31^ (Fig. 1B) associated with the downregulation of α-SMA *vs.* CASMC cultured alone (Fig. 1C,D and Supplementary Fig. 1); these changes describe the induction of the synthetic phenotype of vascular smooth muscle cells^32^. COX-2 expression was induced in CASMC cocultured with platelets (Fig. 1D and Supplementary Fig. 1). The downregulation of α-SMA and upregulation of COX-2 induced by platelets in CASMC were confirmed by Western blot analysis (Fig. 2A–D). In contrast, CASMC COX-1 expression was not affected by their coculture with platelets (Fig. 2C,E).

### Platelets enhanced the migratory capacity of CASMC

**Figure 3:**
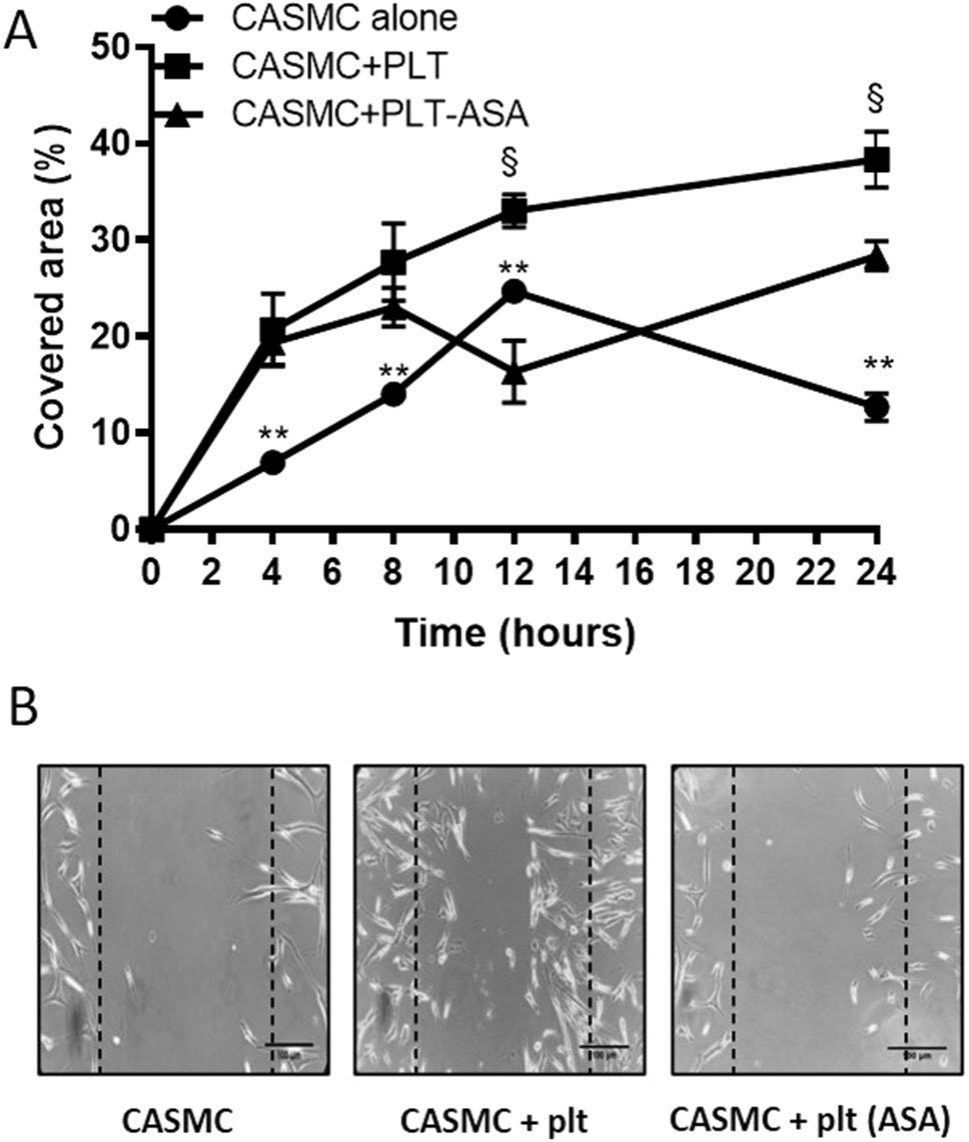
Effects of platelets on CASMC migration. (**A**) Human CASMC (0.8 × 10^5^ cells) were cultured alone or cocultured with human platelets (0.5 × 10^8^) (pretreated or not with Aspirin, ASA, 100 µM) for 20 h and cell migration of confluent cells was evaluated at different time-points (up to 24 h) by scrape wounding assay, using an inverted microscope and a digital camera (original magnification × 10), the dashed lines indicate the starting position of the cell migration occurring toward the center of the wound; % of covered area values are reported as mean ± SEM, n = 3 (separate experiments), the scale bar is of 100 µm. In each experimental condition, % covered area was significantly different (*P* < 0.01) at each time-point versus time 0 (using two-way ANOVA, Dunnett's multiple comparisons test), not shown. At 4 h, **P < 0.01, CASMC alone versus CASMC + PLT and CASMC + PLT-ASA; at 8 h, **P < 0.01, CASMC alone versus CASMC + PLT and CASMC + PLT-ASA; at 12 h, **P < 0.01, CASMC alone versus CASMC + PLT and CASMC + PLT-ASA, ^§^*P* < 0.01, CASMC + PLT versus CASMC + PLT-ASA; at 24 h, **P < 0.01, CASMC alone versus CASMC + PLT and CASMC + PLT-ASA, ^§^*P* < 0.01, CASMC + PLT versus CASMC + PLT-ASA (using two-way ANOVA, Tukey's multiple comparisons test). (**B**) The results of cell migration obtained at 24 h are shown.

Cell migration of CASMC cultured alone or with platelets for 20 h was evaluated by scrape wounding assay^23^. As shown in Fig. 3A,B, platelets enhanced the migration of CASMC. CASMC cultured with platelets that were pretreated with Aspirin (to cause a selective inhibition of platelet-COX-1 activity and TXA_2_ generation) showed a significantly lower migratory capacity versus CASMC cultured with platelets untreated with Aspirin, at 8–24 h (Fig. 3A,B).

### Prostanoid biosynthesis in CASMC-platelet cocultures

**Figure 4:**
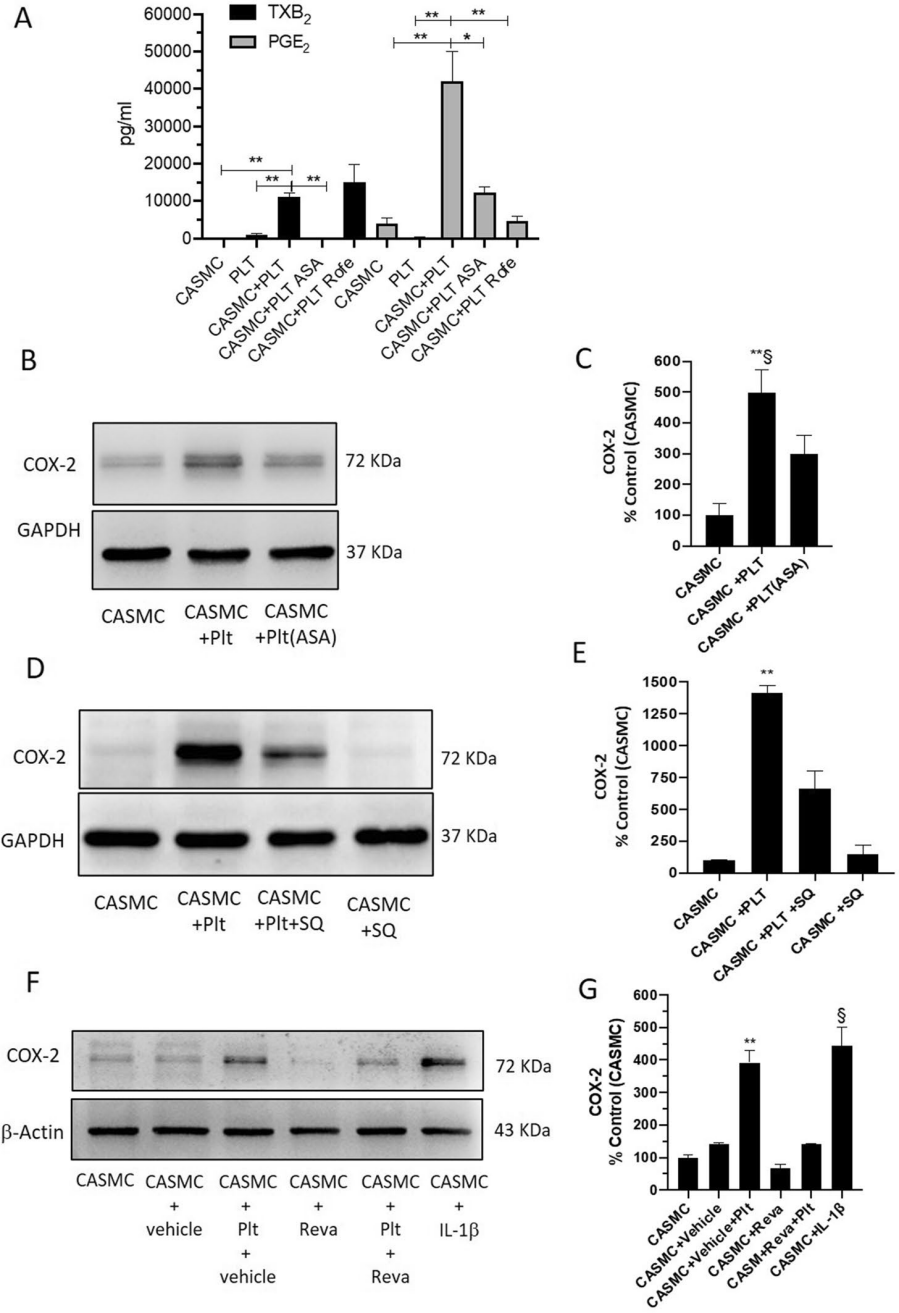
Effects of platelets on prostanoid generation and COX-2 expression of CASMC. (**A**) Levels of PGE_2_ and TXB_2_ in the conditioned medium of CASMC and platelets cultured alone, or cocultured for 20 h, assessed by immunoassays; the effect of Rofecoxib (Rofe, 0.3 µM) or aspirinated platelets (ASA, 100 µM was added during platelet isolation and then washed away) was assessed. **P < 0.01, *P < 0.05; n = 3–4 (separate experiments) (using one-way ANOVA, Tukey's multiple comparisons test). (**B**,**E**) The effect of ASA (100 µM) or of the TP antagonist SQ 29,548 (SQ, 10 µM) on COX-2 expression in cocultures of CASMC and platelets, was assessed by Western Blot. (**F**,**G**) CASMC were cultured alone or cocultured with platelets in the presence of Revacept (Reva, 40 µg/ml) or vehicle (PBS/4% mannitol/1% sucrose) and COX-2 levels were analyzed by Western blot; interleukin (IL)-1β was used as positive control. (**C**,**E**,**G**) COX-2 protein bands were quantified, and the optical density (OD) was calculated using laser densitometry and normalized to the OD of GAPDH or β-actin; data are reported as % of control (CASMC cultured alone), mean ± SEM. (**C**) **P < 0.01 versus CASMC and ^§^*P* < 0.05 versus CASMC + PLT(ASA), n = 3 (separate experiments); (**E**) **P < 0.01 versus all other conditions, n = 4 (separate experiments); (**G**) **P < 0.01 versus all other conditions, except CASMC-IL1β, ^§^*P* < 0.01 versus all other conditions, except CASMC-vehicle-PLT, n = 3 (separate experiments); all analyses were conducted using one-way ANOVA and Tukey's multiple comparisons test.

In platelets cultured alone, TXB_2_ was the primary product of arachidonic acid metabolism (1014 ± 817 pg/ml) (Fig. 4A) while PGE_2_, PGF_2α_, and PGD_2_ were minor products of COX-1 activity (all < 250 pg/ml, not shown). In CASMC cultured alone, PGE_2_ (Fig. 4A) and 6-keto-PGF_1α_ (not shown) were the most abundant prostanoids (3903 ± 3335 and 1310 ± 1271 pg/ml, respectively). In the coculture of platelets and CASMC, PGE_2_ and TXB_2_ were significantly enhanced (Fig. 4A). 6-keto-PGF_1α_ did not significantly change in CASMC-platelet cocultures versus CASMC cultured alone (not shown). To verify the COX-1 or COX-2 origin of the increased biosynthesis of TXB_2_ and PGE_2_ in the coculture of CASMC and platelets, we assessed the impact of the exposure of platelets to Aspirin which was washed away before the incubation with CASMC (to target only platelet COX-1) or Rofecoxib (a selective COX-2 inhibitor). As shown in Fig. 4A, the increase of TXB_2_ detected in platelet-CASMC cocultures was entirely prevented by the treatment of platelets with Aspirin, suggesting that enhanced TXB_2_ derived from platelets. This is further sustained by Rofecoxib inability to affect TXB_2_ production in the coculture (Fig. 4A). Rofecoxib completely prevented the increased production of PGE_2_ measured in platelet-CASMC cocultures, implying that COX-2 induction in CASMC played a central role in enhanced PGE_2_ biosynthesis (Fig. 4A). PGE_2_ levels detected in the coculture medium were significantly reduced when aspirinated platelets were incubated with CASMC (Fig. 4A).

### Effect of aspirin on platelet-induced COX-2 expression in CASMC

As shown in Fig. 4B,C, the pretreatment of platelets with Aspirin attenuated the capacity of platelets to induce COX-2 in CASMC. These results suggest that platelets induced COX-2-dependent PGE_2_ via an Aspirin sensitive mechanism.

### Effect of TXA_2_ receptor (TP) blockage on COX-2 induction in platelet-CASMC coculture

We tested the role of TXA_2_ in the induction of COX-2 in CASMC exposed to platelets. To this aim, we used the selective TP antagonist SQ 29,548. As shown in Fig. 4D,E, TP blockage mitigated the induction of COX-2 in CASMC by the interaction with platelets. These results show that platelet-derived TXA_2_ is the trigger of COX-2 induction in CASMC.

### Effect of the antiplatelet agent Revacept on COX-2 induction in CASMC-platelet cocultures

Vascular smooth muscle cells synthesize and secrete collagen^33^, contributing to the interaction with platelets and their activation. Thus, we tested whether Revacept, which affects the binding of platelets to collagen binding sites^19^, may prevent the induction of COX-2 in CASMC. As shown in Fig. 4F and G, Revacept mitigated platelet capacity to induce COX-2 in CASMC.

### Revacept prevented the increase of systemic TXA_2_ biosynthesis in response to injury in mice

**Figure 5:**
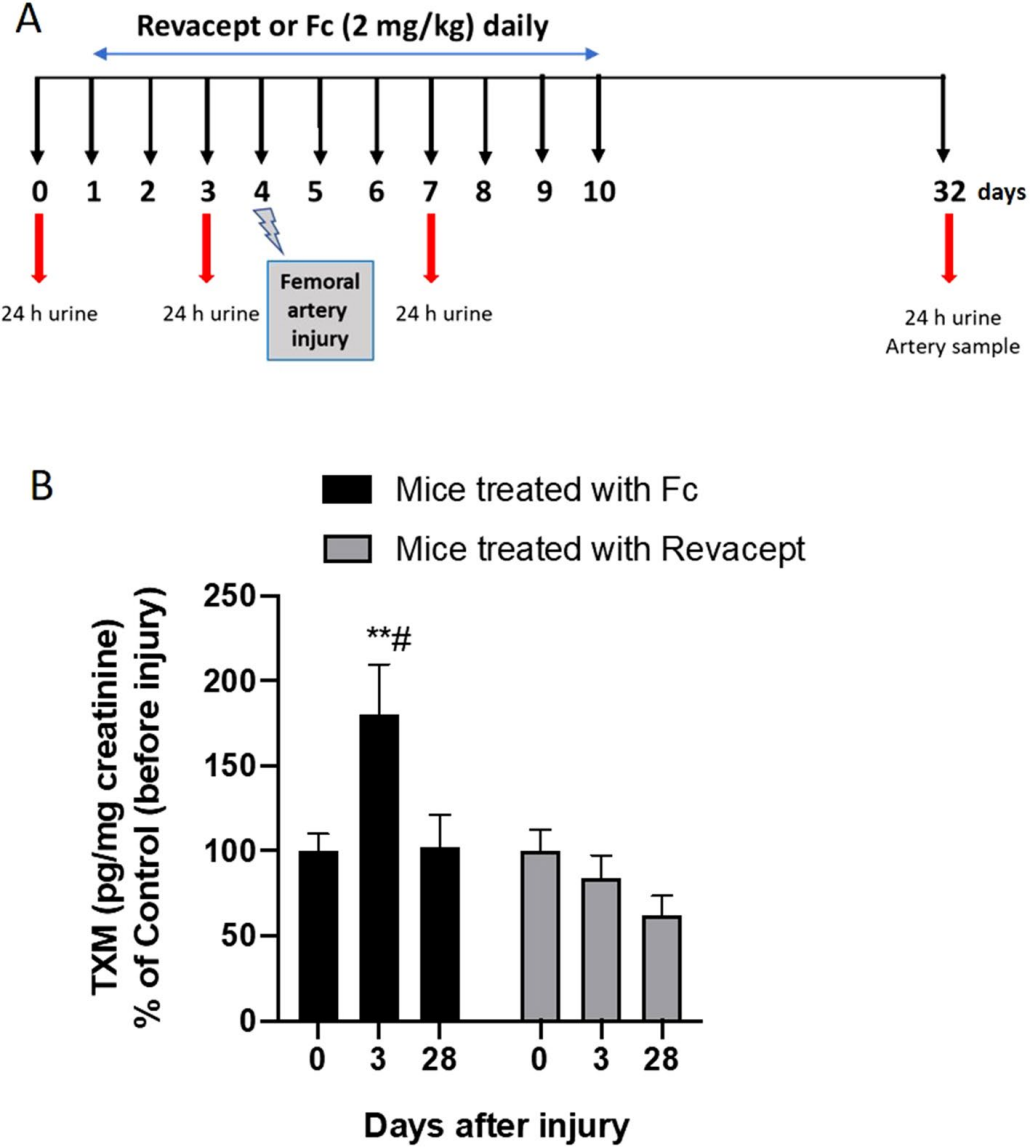
Effects of Revacept administration on urinary levels of TXM in mice in response to injury. (**A**) Study design of Revacept or Fc-control (2 mg/kg/day) administration to mice using a model of neointima formation caused by the damage of femoral arteries via transluminal wire injury. (**B**) Urinary levels of TXM were assessed at different time-points, before (0) and after vascular injury (3 and 28 days) in mice treated with Fc control or Revacept (2 mg/kg/day); data are expressed as mean ± SEM, n = 9–10; **P < 0.01 versus the other Fc conditions, ^#^*P* < 0.05 versus the conditions of Revacept after injury (using mixed-effects analysis for repeated measures data and Tukey's multiple comparisons test).

We used the femoral artery wire injury model^25,26^ in C57BL/6 mice to verify the occurrence of platelet activation in response to vascular damage by assessing the urinary levels of TXM, an enzymatic metabolite of TXB_2_, that is a marker of the systemic biosynthesis of TXA_2_ mainly derived from activated platelets^28,29^. Moreover, we studied whether the administration of Revacept (2 mg/kg/day) to mice prevented the increase of TXA_2_ biosynthesis in response to vascular injury as compared with mice treated with recombinant human IgG1 Fc (2 mg/kg/day) (control group). The experimental design of the study is reported in Fig. 5A.

At baseline, urinary TXM levels were comparable in the two groups of mice (Fc-control: 1.2 ± 0.6 ng/mg creatinine; Revacept: 1.3 ± 0.5 ng/mg creatinine; n = 10) (not shown). As shown in Fig. 5B, in mice treated with Fc-control, the urinary levels of TXM significantly (*P* < 0.05) increased at 3 days after injury versus the values detected before vascular damage. At 28 days after injury (remodeling phase), TXM values were comparable to those measured at pre-injury condition (Fig. 5B). These results show that platelet activation occurs in response to damage because of the endothelial denudation and subintimal component exposure. In contrast, in the remodeling phase, platelet function returns to the baseline condition.

The administration of Revacept, 2 mg/kg daily, prevented the increase of TXM levels (*P* < 0.05) versus Fc control at three days after vascular injury (Fig. 5B).

### Revacept reduced the expression of markers of vascular neointima proliferation and macrophage infiltration

**Figure 6:**
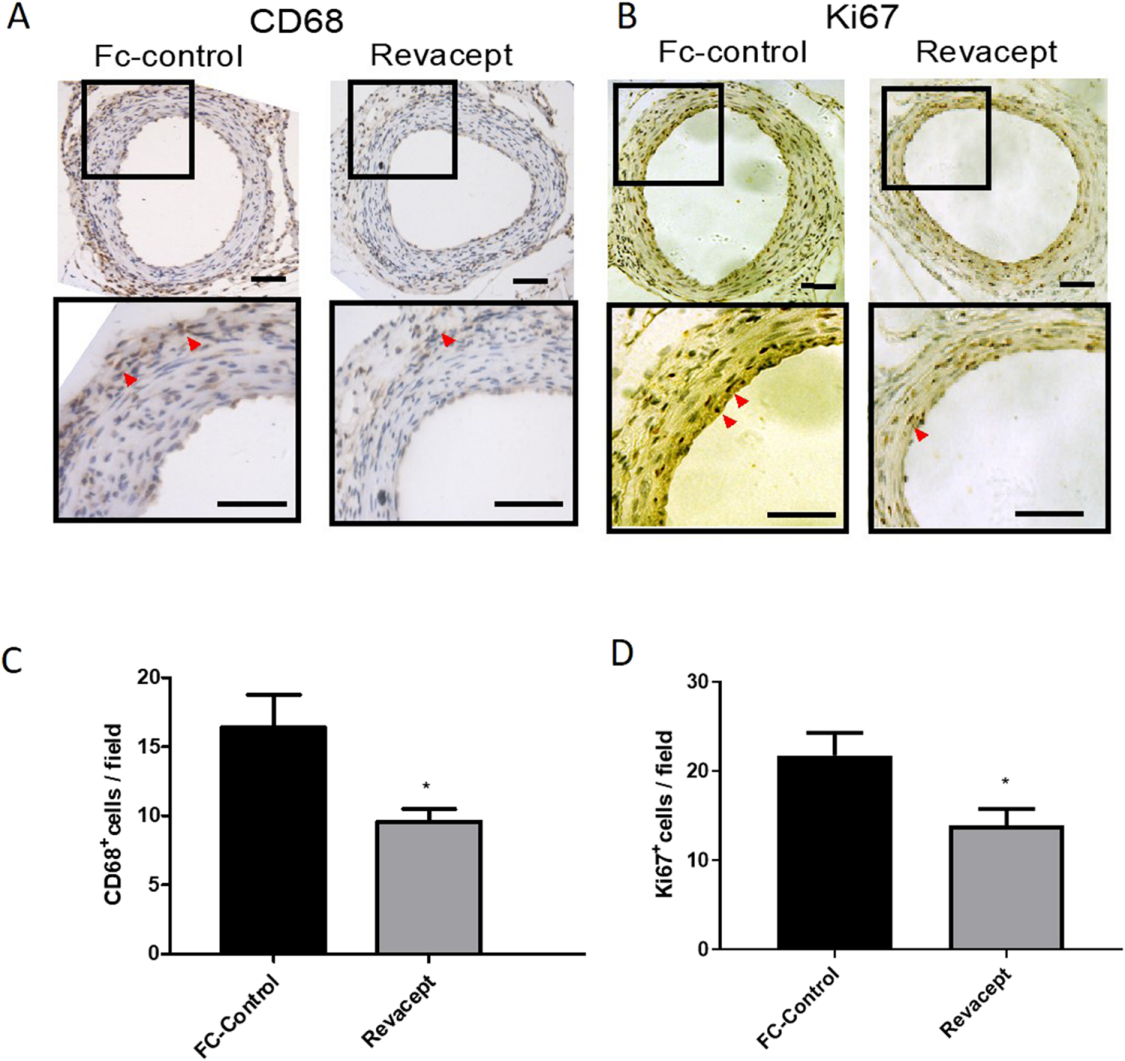
Effects of Revacept administration to mice on CD68 and Ki67 expression in femoral artery sections. (**A**,**B**) Immunohistochemistry of femoral artery sections to assess CD68 and Ki67 at 28 days after surgery in mice treated with Revacept or Fc. (**C**,**D**) CD 68^+^ and Ki67^+^ cells per field are presented as mean ± SEM, n = 4 mice; *P < 0.05 versus Fc-control, (using Student’s t-test). Scale bar = 50 μm.

In the remodeling phase (at 28 days after injury), we studied the expression levels of proteins, which are markers of macrophages infiltration and cell proliferation, such as CD68 and Ki67, respectively, in paraffin-embedded section of femoral arteries. The vascular injury was associated with an increased expression of CD68 (Fig. 6A,C) and Ki67 (Fig. 6B,D). The administration of Revacept mitigated the enhanced expression of these markers (Fig. 6A–D).

Altogether these results show that the inhibition of platelet activation in response to vascular injury by Revacept translates into the prevention of the proliferative and inflammatory events which feature the remodeling phase.

### Revacept reduced vascular neointima formation in response to injury in mice

**Figure 7:**
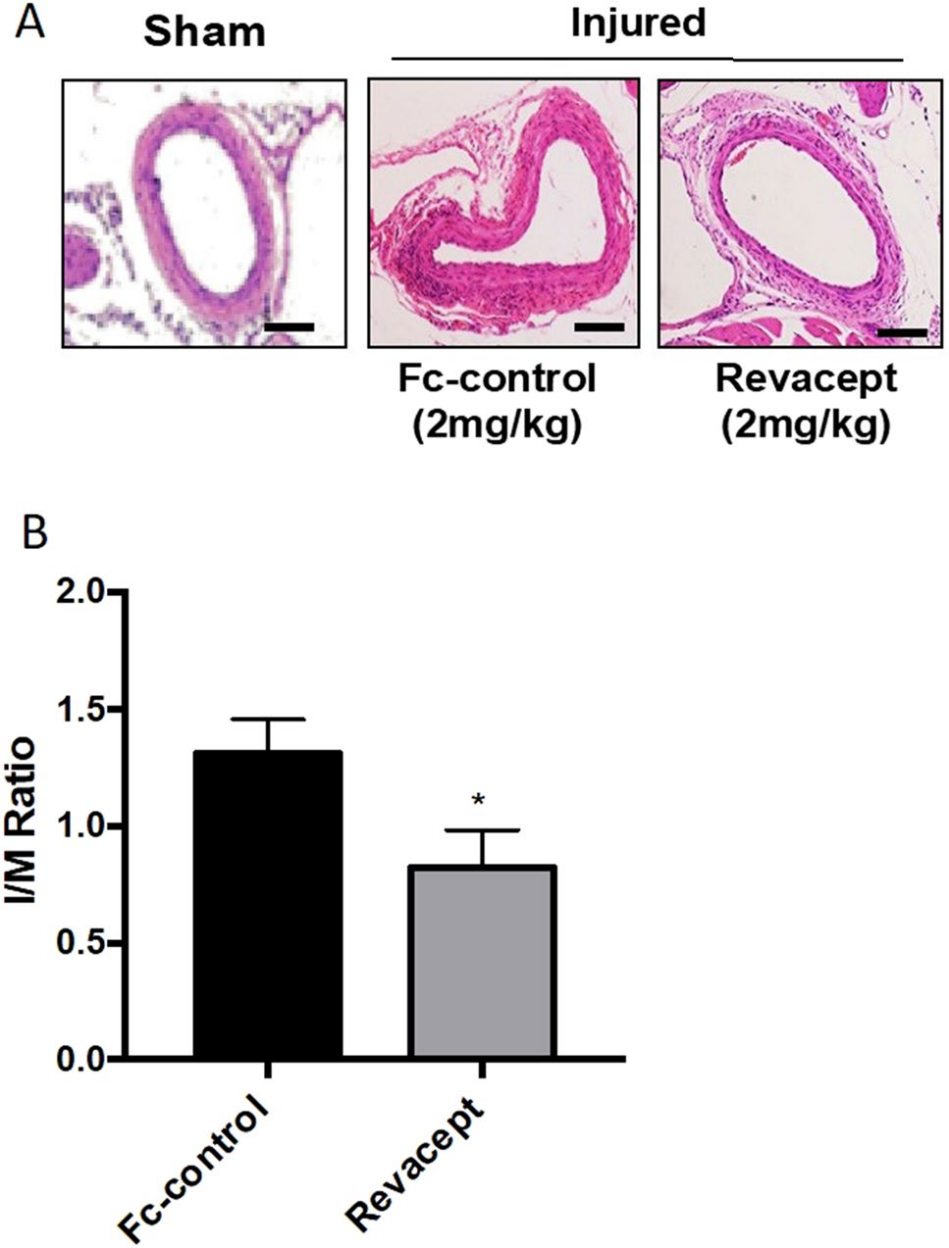
Effects of Revacept administration neointima formation following vascular injury in mice. (**A**) Hematoxylin and eosin staining of cross-sections of injured femoral arteries from mice treated with Fc-control or Revacept (2 mg/kg/day), harvested at 4 weeks after transluminal wire injury. (**B**) Quantification of intima-to-media (I/M) ratio, data are reported as mean ± SEM, n = 6–10; *P < 0.05 versus Fc-control (using Student’s t-test**)**.

In this model of neointima hyperplasia induced by mechanical damage, we evaluated the effect of the administration of Revacept on the arterial media thickness in paraffin-embedded sections of femoral arteries. As reported in Fig. 7A and B, 28 days after arterial injury, Revacept caused a significant (*P* < 0.05) average reduction of I/M ratio by 41.36% versus the Fc-control group.

## Discussion

Platelet activation represents an early response to vascular damage^34^. Platelets adhere to extracellular matrix proteins exposed in the injured vasculature and release numerous soluble mediators, including TXA_2_. It acts in an autocrine or paracrine manner to stimulate adjacent platelets, thus generating more TXA_2_ and amplifying other agonist action^6^. TXA_2_ also enhances lymphocyte and macrophage functions^35–37^ and stimulates the biosynthesis of extracellular matrix proteins^38^. For all these actions, TXA_2_ might play a crucial role in initiating and accelerating intimal hyperplasia development.

In a femoral artery wire injury model^25,26^ in C57BL/6 mice, we assessed the time-course of the systemic biosynthesis of TXA_2_ by measuring the urinary levels of TXM, an enzymatic metabolite of TXB_2_, which is mainly derived from activated platelets^28,29^. Our results show a rapid increase in the systemic biosynthesis of TXA_2_ in response to vascular damage, which returned to pre-injury values at a later phase on neointimal hyperplasia (i.e., 28 days after injury). The enhanced generation of platelet TXA_2_ may induce numerous cellular events that contribute to neointimal hyperplasia. By performing coculture experiments between platelets and CASMC, we have shown that platelet-derived TXA_2_ contributes to COX-2 induction and enhanced PGE_2_ generation by CASMC. We exploited Aspirin capacity to cause an irreversible inhibition of platelet COX-1-dependent TXA_2_ generation, persisting when the drug is washed away. The incubation of CASMC with Aspirin-treated platelets mitigated the induction of COX-2. Comparable results were obtained by TP blockage.

Zhang et al.^12^ have previously shown that COX-2-derived PGE_2_ promotes vascular neointimal hyperplasia in response to mechanical injury. Moreover, COX-2-derived PGE_2_ regulates polarization and directional migration of vascular smooth muscle cells via the activation of the PGE_2_ receptor subtype EP3α/β^12^. Here, we show that platelets enhanced the migration of CASMC; this response was mitigated by the selective inhibition of platelet COX-1 by Aspirin.

Collectively these results suggest that platelets are activated by the interaction with vascular smooth muscle cells and extracellular matrix proteins, such as collagen, and release TXA_2_, which contributes to enhanced COX-2-dependent PGE_2_ biosynthesis. Our findings imply the effectiveness of pharmacological inhibition of platelet TXA_2_ biosynthesis by Aspirin to restrain the development of injury-induced vascular neointimal hyperplasia. However, the results of clinical trials with Aspirin suggest a small reduction in the restenosis after coronary angioplasty but a significant reduction in the frequency of ischemic complications^39^. The weakness of these studies is that different doses of Aspirin were used; furthermore, the drug capacity to cause a virtually complete suppression of platelet COX-1-dependent TXB_2_ biosynthesis was not verified^40^. The possible beneficial effect of low-dose Aspirin in patients undergoing percutaneous coronary intervention through the inhibition of vascular inflammation triggered by activated platelets remains to be verified in appropriate clinical studies.

Aspirin exerts antiplatelet effects by inhibiting TXA_2_‐dependent aggregation responses and amplifying these responses by released ADP^41^. Conversely, Revacept combines inhibitory effects on both collagen-mediated platelet adhesion and subsequent aggregation at the site of vascular injury^19^. These concomitant inhibitory effects on platelet function by Revacept are proper to limit the consequences of vascular damage. We show that the administration of Revacept mitigated vascular neointima hyperplasia in a femoral artery injury mouse model. This effect was associated with reduced vascular expression of markers of macrophages infiltration and cell proliferation. Revacept administration prevented the rapid increase of the systemic biosynthesis of TXA_2_ after vascular injury derived mainly from platelets^9,10^. These data support the hypothesis that platelet activation in response to vascular injury represents an early event contributing to the inflammatory response involved in vascular remodeling.

Interestingly, the interaction of platelets with CASMC in vitro led to morphological changes typical of a synthetic phenotype with downregulation of α-SMA and upregulation of COX-2. These changes were associated with the enhanced migratory capacity of CASMC. Revacept prevented the induction of COX-2 by the interaction of platelets with CASMC.

Collectively our findings sustain the distinct roles of platelets beyond their fundamental participation in primary hemostasis. As previously shown in cocultures with myofibroblasts, platelet-derived TXA_2_ induces morphological changes and enhances their capacity to proliferate and migrate, thus contributing to tissue fibrosis^23^. Moreover, we have shown that the interaction of platelets with colon cancer cells translates into the overexpression of COX-2, a hallmark of malignancy, and the induction of marker genes of epithelial-mesenchymal transition^21^. Inhibition of platelet-cancer cell interactions by Revacept prevented platelet-induced COX-2 induction and changes of epithelial-mesenchymal transition markers^21^.

The main limitation of the present study is that the effect of Revacept on neointimal formation was not compared with Aspirin given alone or co-administered with Revacept. Low-dose Aspirin could cause additional efficacy by preventing the amplification of platelet response induced by other platelet agonists, in addition to collagen. This is an important point that needs to be explored in a specific study.

In conclusion, we show that Revacept, an inhibitor of the binding of platelet collagen receptors (mainly GPVI) to collagen exposed in areas of damaged endothelium^19^, constrains the release of TXA_2_ from activated platelets. These distinct effects played by Revacept make it a promising therapeutic strategy to prevent restenosis in patients with coronary artery disease treated with percutaneous transluminal coronary angioplasty and stent implantation. Importantly, Revacept was found to affect platelet function in the absence of bleeding complications^19^. A phase II randomized, double blind trial (www.clinicaltrials.gov; NCT03312855) is ongoing to assess the efficacy and safety of Revacept in patients undergoing elective percutaneous coronary intervention (ISAR-PLASTER Trial^42^.

## Supplementary information

Supplementary Information.
