# Supplementary material for: The antiplatelet agent revacept prevents the increase of systemic thromboxane A2 biosynthesis and neointima hyperplasia
Source: Sci Rep. 2020 Dec 8;10:21420. doi: 10.1038/s41598-020-77934-x (PMC7722842; doi:10.1038/s41598-020-77934-x)
Supplement: Supplementary file 1 — Supplementary Information. [file 41598_2020_77934_MOESM1_ESM.docx]

**Supplementary material**

**The antiplatelet agent Revacept prevents the increase of systemic thromboxane A_2_ biosynthesis and neointima hyperplasia**

Sara Alberti,^1,2 °^ Qianqian Zhang,^3°^ Ilaria D’Agostino,^1,2°^ Annalisa Bruno,^1,2^ Stefania Tacconelli,^1,2^ Annalisa Contursi,^1,2^ Simone Guarnieri,^2^ Melania Dovizio,^1,2^ Lorenza Falcone,^2^ Patrizia Ballerini,^1,2^ Götz Münch,^4^ Ying Yu,^3^ and Paola Patrignani^1,2^*

^1^Department of Neuroscience, Imaging and Clinical Science and ^2^CAST (Center for Advanced Studies and Technology), “G. d’Annunzio” University, School of Medicine, Chieti, Italy; ^3^ International Peace Maternity and Child Health Hospital of China Welfare Institution, China; ^4^AdvanceCOR GmbH, Martinsried, Germany; ^5^Shanghai Institute for Biological Sciences, Chinese Academy of Science, Shanghai, and Department of Pharmacology, School of Basic Medical Sciences, Tianjin Medical University, Tianjin, China

**Supplementary Methods**

**Assessment of COX-2 and α-SMA by Western blot**

In different experiments of CASMC cultures, we assessed COX-2 and/or α-SMA by Western blot techniques. After verifying that the primary antibodies (see the Methods section in the manuscript text) for the two proteins were of good quality, i.e. specific and had little background, when tested individually, we used two strategies: (i) we incubated together the two antibodies [and a typical blot is shown in Supplemental Figure 1 showing the band of α-SMA (43 kDa) and COX-2 (72 kDa)] or (ii) we cutted the membrane horizontally at 50 kDa and we incubated the two membranes with the specific antibodies against α-SMA or COX-2 (Supplementary Figures 2-5). The secondary antibodies were incubated after washing the membrane with TTBS.

For the assessment of loading control of the Western blot, we used β-actin (43 kDa) or GAPDH (37 kDa) which have ubiquitous expression. The membranes cutted at 50 kDa (containing proteins with molecular weight from 50 to 25 kDa) were extensively washed in TTBS, then, they were incubated with the primary (see the Methods section on the manuscript) and secondary antibodies (see the Methods section on the manuscript) to detect the β-actin (specific immunoband at 43 kDa), then the membranes were extensively washed with TTBS and re-probed with the primary and secondary antibodies to detect the GAPDH specific immunoband (at 37 kDa).


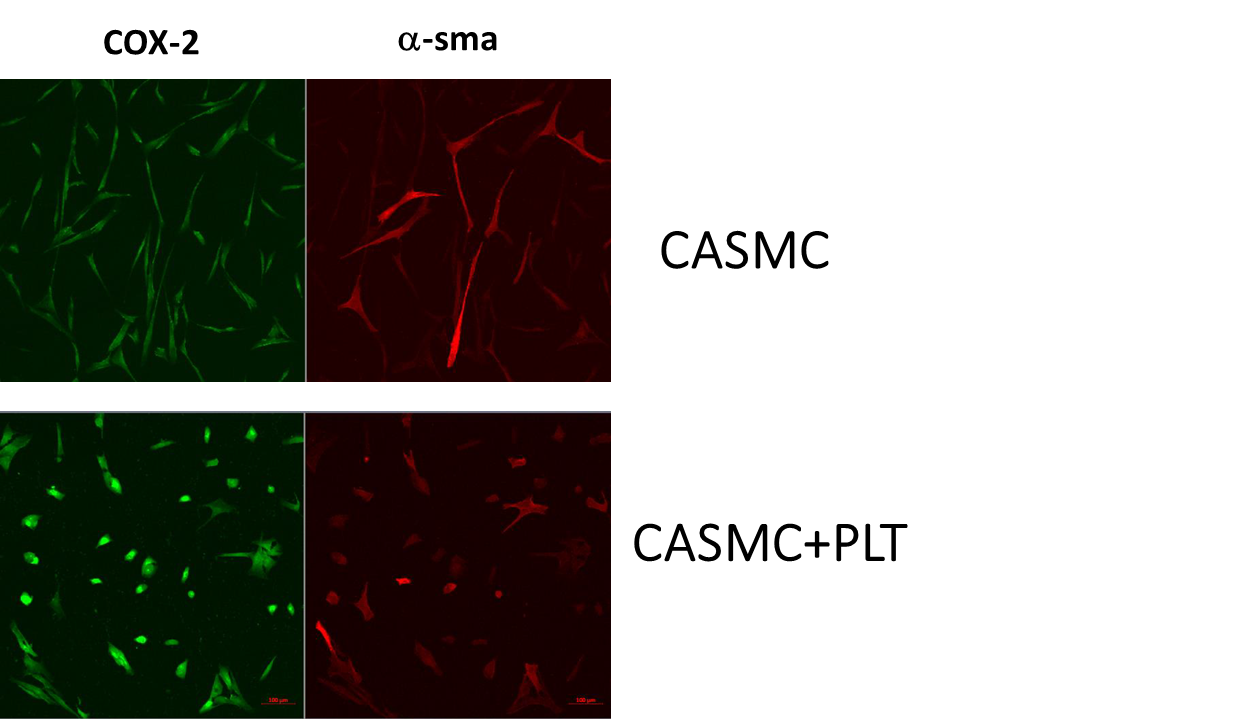


**Supplementary Figure 1.** Confocal images of the single green channel (COX-2 fluorescent signal) and the single red channel (α-SMA fluorescent signal) related to merge images reported in Figure 1 C and D. Human CASMC (0.8x10^5^ cells) were cultured alone or cocultured with human platelets (0.5x10^8^).

**Supplementary Figures**


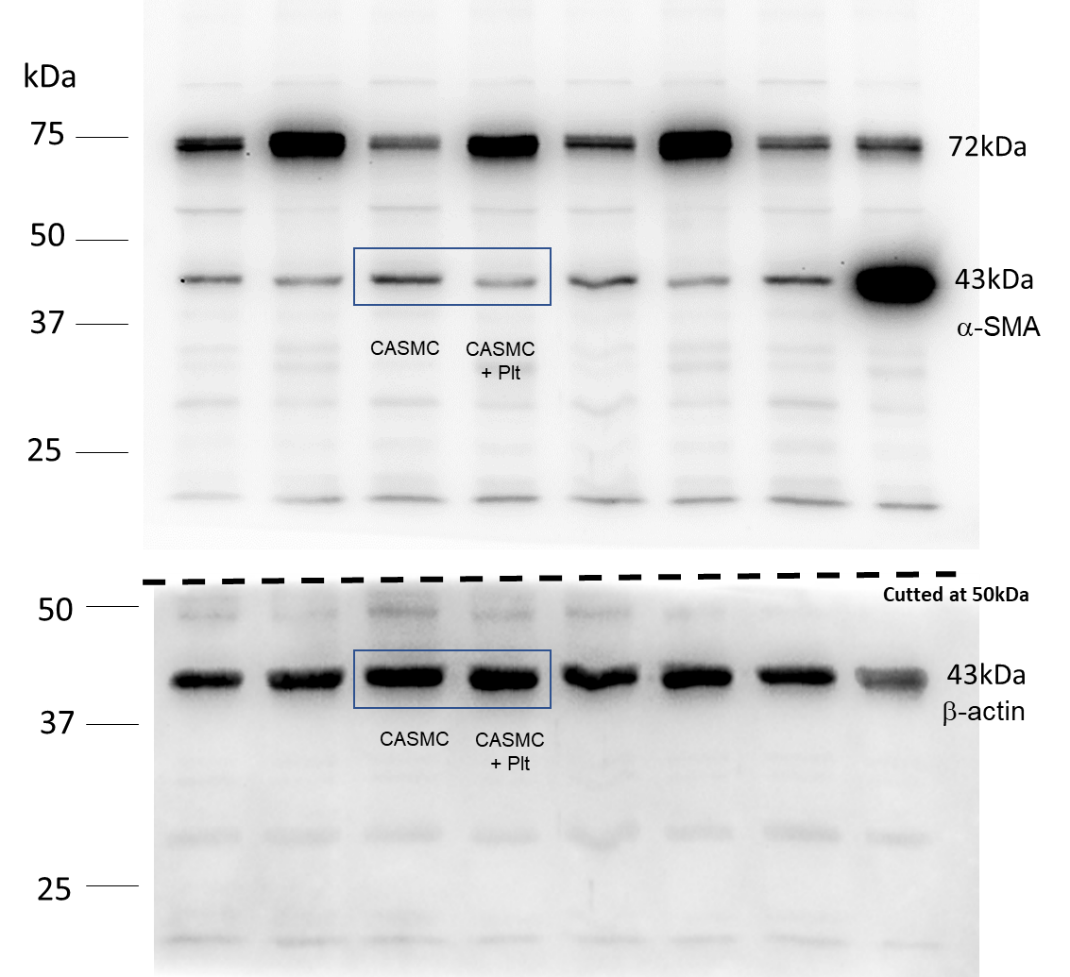


**Supplementary Figure 2. Uncropped gels of Figure 2A.** The upper Western blot membrane was incubated with the primary and secondary antibodies to detect the α-SMA specific immunoband (at 43 kDa); the band detected at 72kDa was COX-2 protein since the membrane was also incubated with the primary and secondary antibodies to COX-2. Then, the membrane was cutted at 50 kDa, washed with TTBS and the lower membrane was re-probed with the primary and secondary antibodies to detect the β-actin specific immunoband (at 43 kDa).


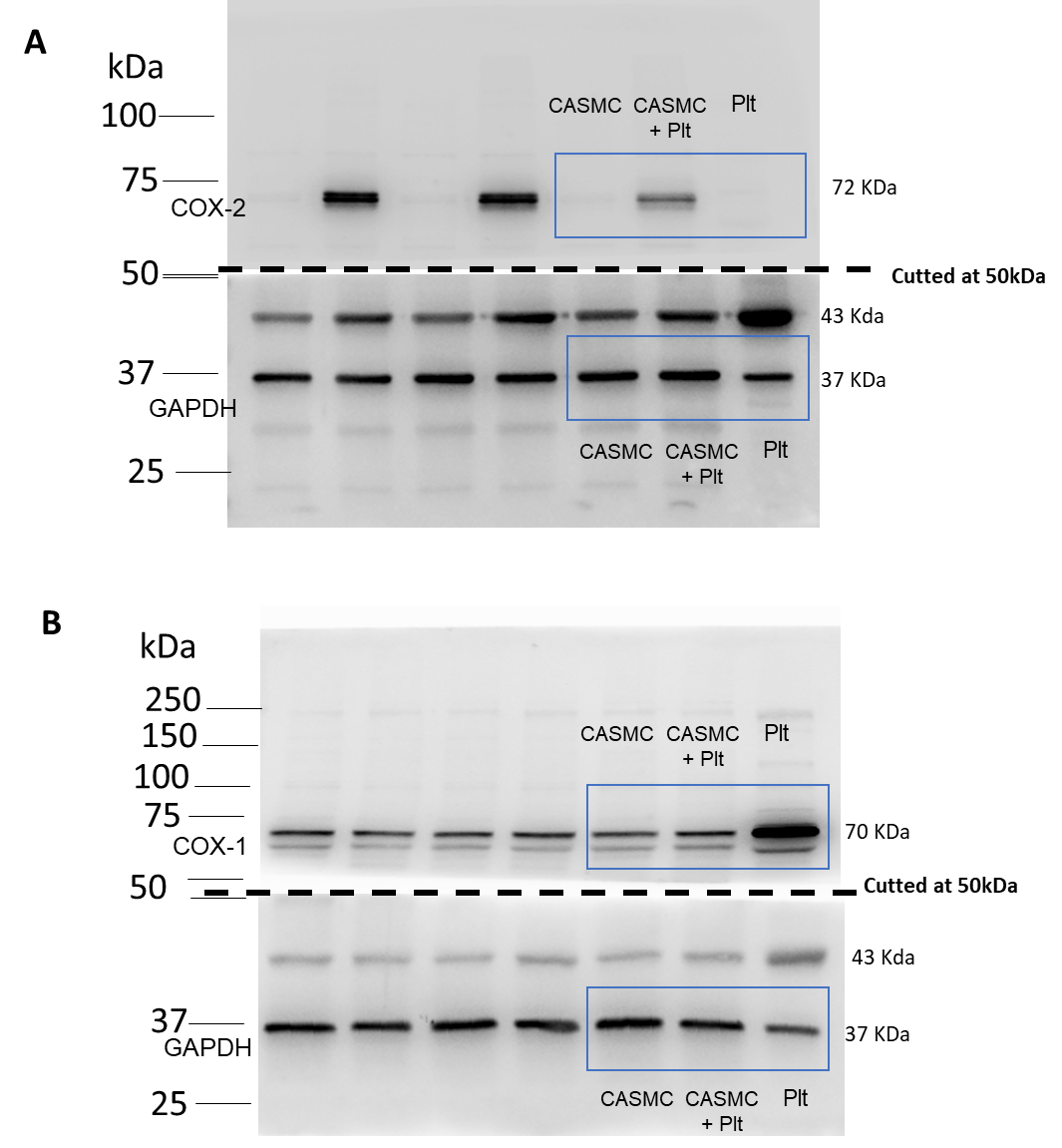


**Supplementary Figure 3. Uncropped gels of Figure 2C.** The Western blot membranes for COX-2 **(A)** and COX-1 **(B)** analysis were cutted at 50kDa and incubated with the primary and secondary antibodies to detect the COX-2 (**panel A**, upper membrane) and COX-1 (**panel B**, upper membrane). In the **panels A** and **B**, the lower membranes (containing proteins with molecular weight from 50 to 25 kDa) were incubated with the primary and secondary antibodies to detect the β-actin specific immunoband (at 43 kDa), then the membranes were extensively washed with TTBS and re-probed with the primary and secondary antibodies to detect the GAPDH specific immunoband (at 37 kDa).


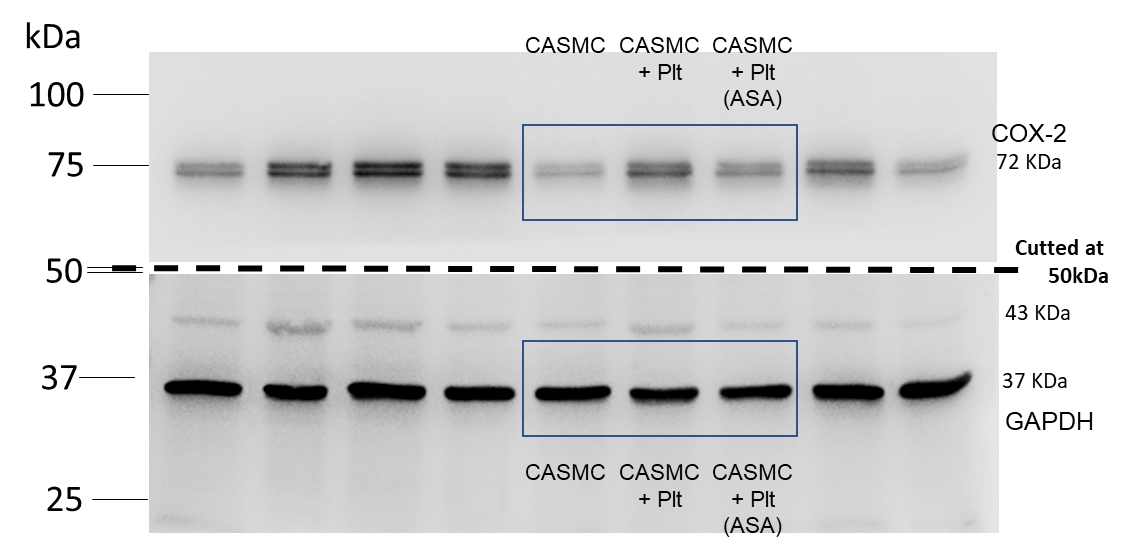


**Supplementary Figure 4. Uncropped gels of Figure 4B.** The Western blot membrane was cutted at 50kDa. The upper membrane was incubated with the primary and secondary antibodies to detect the COX-2 (72 kDa). The lower membrane (containing proteins with molecular weight from 50 to 25 kDa) was incubated with the primary and secondary antibodies to detect the β-actin specific immunoband (at 43 kDa), then the membrane was extensively washed with TTBS and re-probed with the primary and secondary antibodies to detect the GAPDH specific immunoband (at 37 kDa).


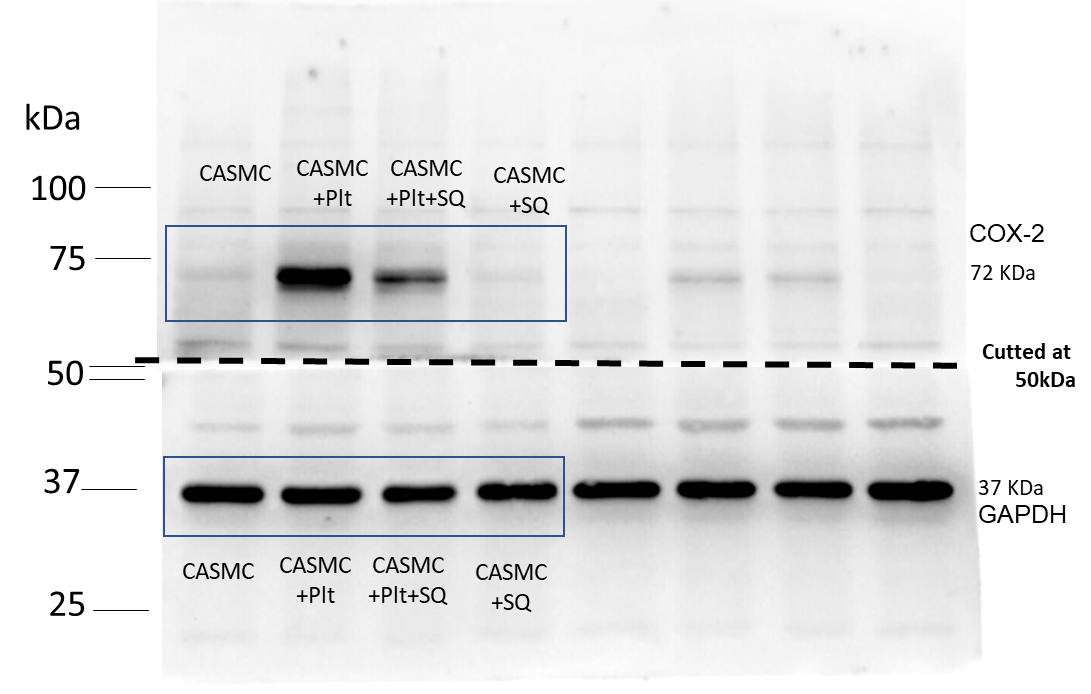


**Supplementary Figure 5. Uncropped gels of Figure 4D.** The Western blot membrane was cutted at 50kDa. The upper membrane was incubated with the primary and secondary antibodies to detect the COX-2 (72 kDa). The lower membrane (containing proteins with molecular weight from 50 to 25 kDa) was incubated with the primary and secondary antibodies to detect the GAPDH specific immunoband (at 37 kDa).


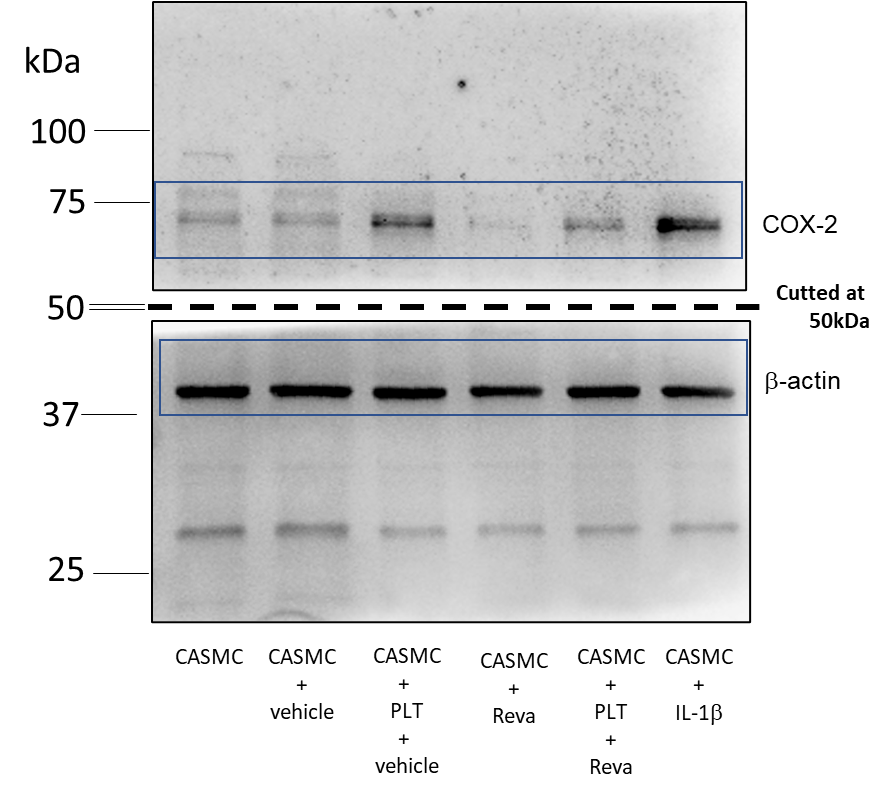


**Supplementary Figure 6. Uncropped gels of Figure 4F.** The Western blot membrane was cutted at 50kDa. The upper membrane was incubated with the primary and secondary antibodies to detect the COX-2 (72 kDa). The lower membrane (containing proteins with molecular weight from 50 to 25 kDa) was incubated with the primary and secondary antibodies to detect the β-actin specific immunoband (at 43 kDa).
